# Exploring Collective Dynamics in Communication Networks

**DOI:** 10.6028/jres.107.016

**Published:** 2002-04-01

**Authors:** Jian Yuan, Kevin Mills

**Affiliations:** National Institute of Standards and Technology, Gaithersburg, MD 20899-0001

**Keywords:** cellular automata, collective dynamics, complex system, congestion control, emergence, communication networks, long-range dependence, modeling and simulation, network traffic

## Abstract

A communication network, such as the Internet, comprises a complex system where cooperative phenomena may emerge from interactions among various traffic flows generated and forwarded by individual nodes. To identify and understand such phenomena, we model a network as a two-dimensional cellular automaton. We suspect such models can promote better understanding of the spatial-temporal evolution of network congestion, and other emergent phenomena in communication networks. To search the behavior space of the model, we study dynamic patterns arising from interactions among traffic flows routed across shared network nodes, as we employ various configurations of parameters and two different congestion-control algorithms. In this paper, we characterize correlation in congestion behavior within the model at different system sizes and time granularities. As expected, we find that long-range dependence (LRD) appears at some time granularities, and that for a given network size LRD decays as time granularity increases. As network size increases, we find that long-range dependence exists at larger time scales. To distinguish effects due to network size from effects due to collective phenomena, we compare congestion behavior within networks of selected sizes to congestion behavior within comparably sized sub-areas in a larger network. We find stronger long-range dependence for sub-areas within the larger network. This suggests the importance of modeling networks of sufficiently large size when studying the effects of collective dynamics.

## 1. Introduction

As the Internet expands in size (from millions to billions of nodes) and in capabilities (from mainly file-transfers and web traffic to an increasing proportion of streaming multimedia traffic), network researchers propose and investigate new protocols and control algorithms to provide assured quality of service and improved network utilization. In most cases, such investigations are motivated and evaluated by small-scale simulations and experiments while ignoring effects from collective behavior that could emerge in a large network. Emergent phenomena are often identified when the behavior of an entire system appears more coherent and directed than the behavior of individual parts of the system. Such phenomena arise in the study of complex systems, where many parts interact with each other and where the study of the behavior of individual parts reveals little about system-wide behavior [1]. Emergent phenomena might significantly influence the performance of proposed new protocols and control algorithms. We aim to identify and understand the source and effects of emergent phenomena in large networks. In this paper, we investigate specifically congestion behavior in a network at various sizes and time scales.

Our work exists within an active field of investigation where researchers attempt to understand and control the dynamic behavior of existing networks. For example, past work on the performance of congestion-control algorithms for end nodes in the Internet [2] identified important effects on system-wide congestion arising from the retransmission behavior of individual nodes. Recently, researchers have begun to investigate mathematical approaches to characterize collective behavior in large networks. For example, Liu et al. [3] propose using stochastic differential equations to describe the behavior of flows and queues, with passage to more tractable systems of ordinary differential equations by taking expectations. The coupled ordinary differential equations can be solved numerically. Similar approaches, based on fluid-flow models, are also being investigated through simulation [4]. Innovative ideas such as these begin to probe the ability of abstract mathematical techniques to characterize collective behavior in large networks.

As an alternative to these more abstract mathematical approaches, some researchers represent large networks in terms of discrete-event simulations of individual nodes deployed in specific topologies. Such models attempt to capture the detailed behavior in individual nodes to ensure that significant effects from protocols and protocol interactions will not be overlooked by the coarser granularity used to construct more abstract models. The advantages of simulation models include the ability to represent the time-varying behavior of a network and to capture complex behavioral details, which might have significant effects on global network behavior. Further, it is always possible to construct discrete-event models for specific protocol mechanisms, while it might prove impossible to develop tractable mathematical models for such mechanisms. Unfortunately, detailed discrete-event simulation models consume substantial CPU time, much of which may be devoted to modeling behavior irrelevant to the phenomena being investigated. Several researchers are working on speeding up discrete-event simulations, through parallelization and other techniques, in order to enable realistic simulation of networks containing from 10^4^ to 10^6^ nodes [5, 6].

We suspect that an interesting space of informative models may exist somewhere between manageable, abstract mathematical models, and detailed, computationally intensive simulations. A growing interest in the study of complex systems has led networking researchers to consider applying a number of mathematical techniques from physics to characterize collective dynamics in large networks [7–11]. One such technique applies a cellular automaton (CA) [12, 13] to identify and characterize emergent properties, such as congestion, in networks [14]. We suspect such models can promote better understanding of the spatial-temporal evolution of network congestion, and other emergent phenomena.

In this paper, we use a two-dimensional CA to investigate congestion behavior in networks of varying size, and over varying time scales. The CA stimulates collective dynamics in a network using abstract mathematical representations of individual nodes. To search the behavior space of our CA, we study dynamic patterns arising from interactions among traffic flows routed across shared network nodes, as we employ various configurations of parameters and two different congestion-control algorithms. We focus on the relationship between network size and time scale. Specifically, we characterize the correlation in congestion behavior within the model at different system sizes and time granularities. Among other things, our results suggest the importance of modeling (or measuring) networks of sufficiently large size when studying the nature of collective dynamics. The paper is presented in five sections. Section 2 outlines our motivation, provides highlights of our methodology, and describes our computational model, including input process, control algorithms, and routing. We outline our experiments, and show our simulation results in Sec. 3. Section 4 discusses the significance of our results. We discuss possible future work in Sec. 5, and then present concluding remarks in Sec. 6.

## 2. Motivation, Methodology, and Model

We seek to understand what will happen when many network connections are active simultaneously. We suspect that some collective effects may appear within networks with a size beyond some determinable threshold. Our concern is to understand the global implications of such collective effects, rather than to discern cause and effect relationships introduced by specific control algorithms and associated protocols.

Research into emergent phenomena in physical systems shows that collective effects tend to arise only when many local interactions occur over a wide space, such as might be possible in a large network where dynamic behavior can be transmitted through the nodes. The transmission of dynamic behavior can evolve into correlated patterns. Alternatively, large networks can be viewed as dissipative dynamic systems, where a driving force (injecting packets) determines the strength of local interactions. Increasing the driving force impels the system toward stronger coupling among interactions. As the driving force reaches a certain critical strength, we can expect the interactions to merge into a coherent behavior, which cannot be simply inferred from the individual behavior of components. The resulting coherent behavior should be observed as spatial-temporal dynamic patterns over the whole system. We think that this view of system dynamics might prove applicable in large communication networks, such as the Internet. Further, it is possible that feedback control mechanisms, as implemented in such networks, might play a pivotal role in dynamically maintaining both coherence and efficiency of system state by preventing resource saturation due to extreme congestion.

### 2.1 Methodology

**Fig. 1:**
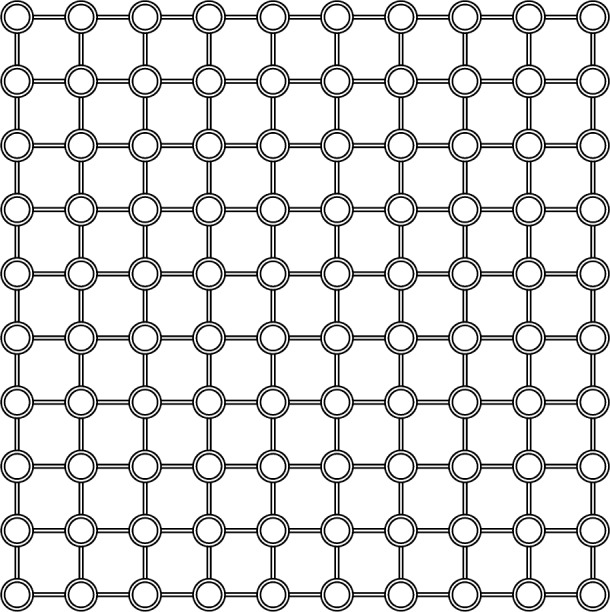
The 2-D CA model with system size *L* = 10.

To investigate these ideas, we propose to model a large network as a cellular automaton (CA), where the behavior of each cell captures some important details related to network protocols. Specifically, our model maintains the individual identity of packets in order to reproduce the “ripple effect” [3] seen in real network connections. Our model also simulates feedback-control regimes for each connection in order to respond to variations in congestion over time and space. We provide two alternative forms of feedback control: connection-admission control (CAC) and feedback transmission-control protocol (TCP). On the other hand, we resort to some extreme simplifications, including a regular grid topology composed of homogeneous links and nodes (see Fig. 1), in order to achieve a sufficiently large model with well-understood parameters. Further, to ensure that no correlation arises from the input traffic itself, we use homogeneous on-off Poisson processes to model the behavior of traffic sources.

Our CA comprises a discrete, dynamic system composed of a set of cells arranged in a regular, spatial lattice. The state of a cell at a given time step depends only on its own state and the states of its neighbors at the previous time step. The cells update synchronously and in parallel. Thus, the entire CA state advances in discrete time steps. Global behavior results from multiple interactions in the evolution of states in all the single cells. As a result of the discrete time model, we can measure the entire CA state, or any portion of interest, at each time step. In our results, we include at least 2000 data points in each time series that we analyze. For our coarsest time granularity, where we aggregate 1000 time steps, this means that we must run the model for two million time steps. Such measurements produce a time series that can be graphed, and that can also be analyzed using a wide range of suitable statistical techniques.

Our main approach to analyze time-series employs power-spectral analysis [24]. We envision the system state as a signal generated over time by the system. Power-spectrum graphs describe the frequency dependence in the signal. The power spectrum is the Fourier transform of the autocorrelation function of a time series. Of particular interest to us is the appearance of 1/*f* noise, which provides a clear indication of some sort of collective effect, or cooperative phenomenon, present in the process that generates the signal [22]. “1/*f* noise” is a type of noise whose power spectrum as a function of frequency (*f*) behaves like: *P*(*f*) = 1/*f ^a^*, where *a* is between 0 and 2. Such noise is often associated with the presence of a complex system, defined as a system that exhibits large variability in its behavior due to strong dynamic interactions among its parts. In contrast to a complex system, an ordered system exhibits regular behavior due to deterministic interactions among its parts, and a disordered system exhibits behavior that can be characterized on a statistical basis because its parts interact rarely [22]. In fact, the presence of 1/*f* noise is often cited in the literature as an indication of emergent phenomena. Further, the power spectrum of 1/*f* noise exhibits ample energy in low frequency components. This suggests the dominant autocorrelation in the signal exists over the long term, which also indicates long-range dependence.

### 2.2 Modeling a Network With Cellular Automata

**Fig. 2:**
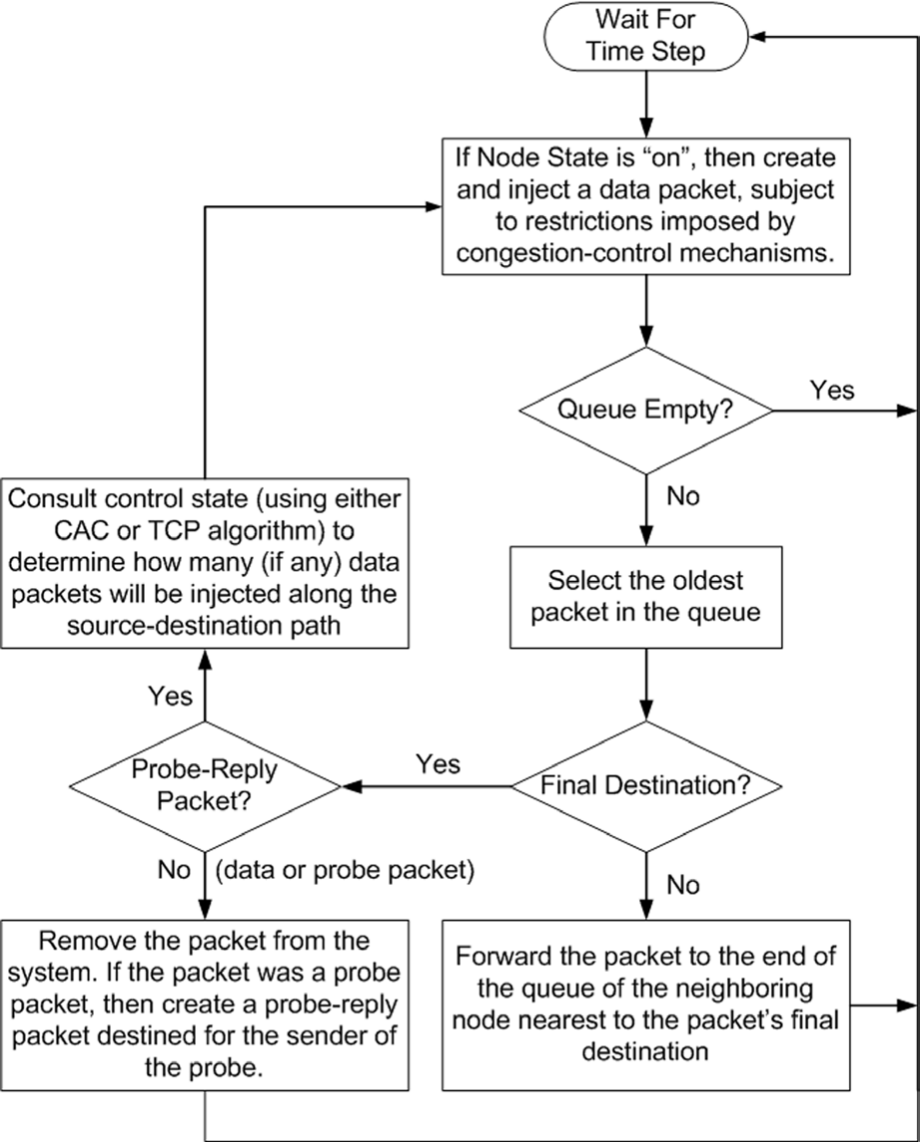
General processing within a node at each time step.

The computational model used here represents a network as a CA lattice with *L*×*L* cells (Fig. 1) in which each cell corresponds to a node with four nearest neighbors (we interconnect boundary nodes, as necessary). Other researchers have proposed similar network models [8, 9, 15, 16]. In our model, each node, which can store and forward packets traveling between source-destination pairs, maintains a queue of unlimited length, where arriving packets are stored until they can be processed. Each node can act both as a source and destination for traffic. In effect, each cell in our model can be thought to represent a host that sends and receives traffic and a router that forwards traffic. The queue length at each node represents the state of the cell. Figure 2 provides a schematic diagram of the node-specific behavior of each CA cell at each time step.

### 2.3 Node as a Cell

At each time step, each node: (1) evaluates the traffic-generation restrictions and congestion-control status and, if indicated, places a new data packet at the end of its own queue, (2) selects a packet from the front of its queue (if a packet is present), (3) selects the next hop (unless the packet has reached its destination node), and (4) forwards the packet to the end of the queue within the next-hop node. If the packet at the front of the queue has reached its destination, then the node consumes the packet. However, if the consumed packet is an incoming request for which a reply is indicated, then the node will place a reply packet at the end of its queue. Different choices are possible to model the input process, the congestion-control algorithm, and the routing. A discussion of each of these issues follows.

#### 2.3.1 Input Process

In this paper, each node models traffic generation by “on/off” periods, which alternate between wake and sleep. When awake, and if the congestion-control state permits, the node adds a data packet to the end of its queue during each time step. At the beginning of each “on” period, a node randomly selects (uniform distribution) a destination node from among all other nodes in the lattice. Each packet generated during the same “on” period has the same destination address. When sleeping, the node generates no new data packets. On/off periods provide a convenient model of user behavior. In the simulation reported here, the wake and sleep period durations for each source are taken to be exponentially distributed with parameters *λ*_on_ and *λ*_off_. Thus, the transitions between state “on” and state “off” form a memoryless (i.e., uncorrelated) process. While we do not assert that real users exhibit memoryless behavior, we aim to investigate long-memory behavior that results from collective interactions; therefore, we must eliminate correlations in the behavior of individual traffic sources. The input processes of different nodes are taken to be independent from each other.

#### 2.3.2 Congestion-Control Algorithms

Our model contains the possibility of three choices for congestion-control algorithm: (1) open-loop, (2) connection-admission control (CAC), and (3) feedback transmission control protocol (TCP). Only one of these algorithms can be used for a given experiment. In the case of the open-loop algorithm, we can change the average durations of state “on” and state “off” to control the network workload. There is no feedback in this case. An open-loop approach does not model reality very well because the goal of a network is to transmit packets between source-destination pairs while attempting to assure some minimally acceptable quality of service. Even for best-effort service (i.e., where the network provides no guarantees about service quality) [2], a user will not wait indefinitely in the face of long packet-transmission times that occur in a very congested network. So feedback control is important for networks, and we model two different approaches.

In one approach, we use a connection-admission control (CAC) algorithm. CAC requires a source to send a probe packet at the beginning of each “on” state. Upon receiving this probe packet, the destination node returns a probe-reply packet to the source. Upon receiving the probe-reply packet, the source node determines the round-trip time, *RTT*, and then normalizes *RTT* with respect to the distance between the source and destination. If the normalized *RTT, Nrtt*, falls below a threshold, *Drtt*, then the source sends a data packet at each time step during the “on” state. If the *Nrtt* exceeds Drtt, then the source sends another probe packet. Upon receiving the probe-reply, the source repeats its *RTT* assessment.

While the CAC algorithm tests congestion state along the source-destination path prior to injecting a data-packet flow, the flows themselves exhibit fixed inter-packet spacing. Inter-packet spacing within a single TCP flow, however, has been observed to exhibit its own distinguishing variability, which appears as structured behavior on a short time scale. Such variability in TCP flows is likely attributable to the feedback-control mechanisms of TCP [17, 18], though this remains a topic of ongoing study. Other studies have demonstrated that TCP results in interesting dynamics at small time scales [19, 20]. To account for the significant behavior of feedback control, we included within our model a modified version of TCP.

Our TCP model includes some limiting assumptions. Each node contains an unlimited buffer, so no packets will be lost and retransmitted. Instead we only model the effect that congestion losses would have on TCP flow-control mechanisms. We do this by comparing the normalized *RTT, Nrtt*, for each received acknowledgment (ACK) against a threshold, *Drtt*. We also ensure that the receiver’s advertised flow-control window [2] does not constrain the sending rate. Our modified TCP model does include a slow-start and congestion-avoidance algorithm, which is described as follows.

For every ACK, if the *Nrtt* exceeds the *Drtt*, then we set the slow-start threshold to 1/2 the congestion window and set the congestion window to one. Otherwise, if the congestion window is below the slow-start threshold, we increment the congestion window by one. Once the congestion window exceeds the slow-start threshold, we increment the congestion window by the inverse of the congestion window. This procedure simulates the effects of the transition in TCP between the congestion phase, the slow-start phase and the congestion-avoidance phase. At each time step, a source node injects a data packet, up to the limits of its congestion window.

#### 2.3.3 Routing

As with input processes and congestion-control algorithms, we can choose different routing strategies for our model. For our experiments, the objective of routing is to minimize the delay for each packet by forwarding it along the shortest path between source-destination pairs. To select the proper next-hop along which to forward a packet, the forwarding node computes (using the approach of Fuks and Lawniczak [15]) the distance from each of its four neighboring nodes to the packet’s destination node. Then the packet is forwarded to the neighboring node nearest to the destination. When multiple neighboring nodes prove equidistant from the destination, then one of the candidate nodes is selected randomly with uniform probability. After placing the packet at the end of the queue in the selected node, the model increments a throughput counter associated with the corresponding outgoing link.

## 3. Simulation Results

In this section, we study the behavioral properties of our model under two congestion-control algorithms. An important parameter in our algorithms is *Drtt*, which can be used to control the quality of service in packet-delivery time, and also the network load. We performed some simulations to assess the effect of *Drtt*, and observed that, for a fixed network size and fixed *λ*_on_ and *λ*_off_, packet delivery time and network congestion increases with *Drtt* up to some bound. Further, for several fixed values of *Drtt*, we observed that the distribution of packet-delivery times seem to follow a lognormal distribution, as observed elsewhere [16, 21]. From these results (not fully described here) we decided to set *Drtt* at 50 time steps, a value that permitted congestion to build up within the network, and thus enabled us to study the collective behavior of our congestion-control algorithms. Unless otherwise indicated, all simulations discussed here were run with *λ*_on_ = 100, *λ*_off_ = 500, *Drtt* = 50, and with the TCP congestion-control algorithm. We begin by considering the effects of time scale on queue sizes in an individual node.

**Fig. 3:**
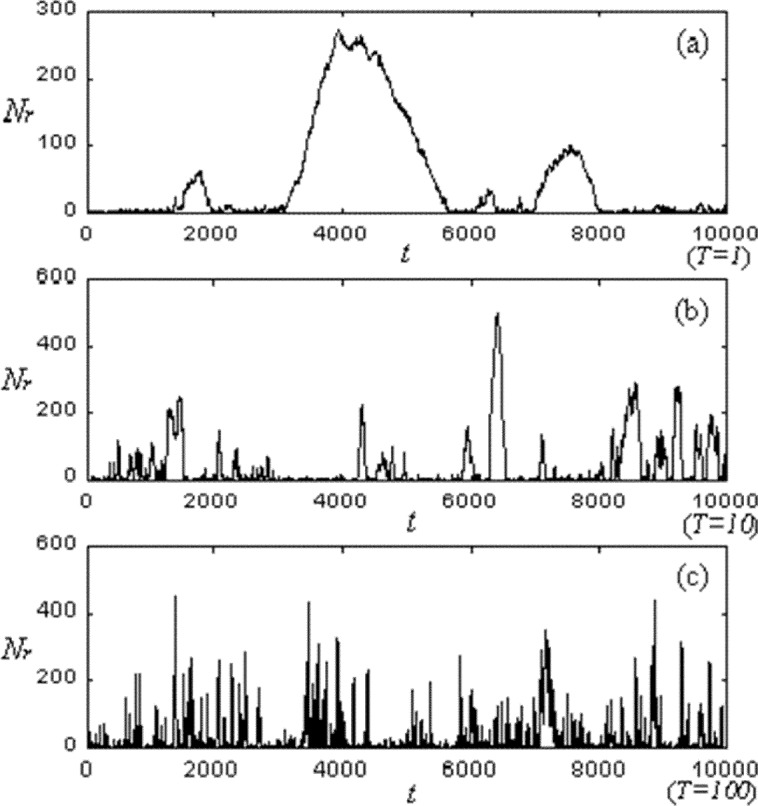
Time series of queue length (*Nr*) at three time granularities *T* = 1, 10, and 100. The total time shown on this, and similar graphs, is equal to *T*×*t*, the sample interval size (*T*) multiplied by the number of sample intervals (*t*).

Packet-switched networks, which route messages hop-by-hop over multiple intermediate links and routers between sources and destinations, can be viewed as a mesh of single-server queues, where each queue acts as a memory element. For purposes of illustration, Fig. 3 shows the time series of the queue length, *Nr*, for a typical node of the CA lattice at three time granularities *T* = 1, 10, and 100, where *T* defines the interval with which we sample the system state. (The abscissas on all our graphs depict the number of sample intervals, *t*, so the total number of CA time steps represented in any particular graph is equal to *T*×*t*). At the shortest sample interval, *T* = 1, the queue length changes smoothly because the memory introduces a correlation. As expected, as the sample interval *T* increases, the memory becomes weaker and the correlative structure is diminished. Since each queue exists within a network of queues, we expect correlation in queue size to be influenced by memories of neighboring queues. This influence appears as a kind of spatial-temporal information, which is difficult to discern by observing a single queue. Before we consider a more suitable metric to measure spatial-temporal memory, we discuss another measure of interest, the output process of an individual node, as an alternative to queue size.

**Fig. 4:**
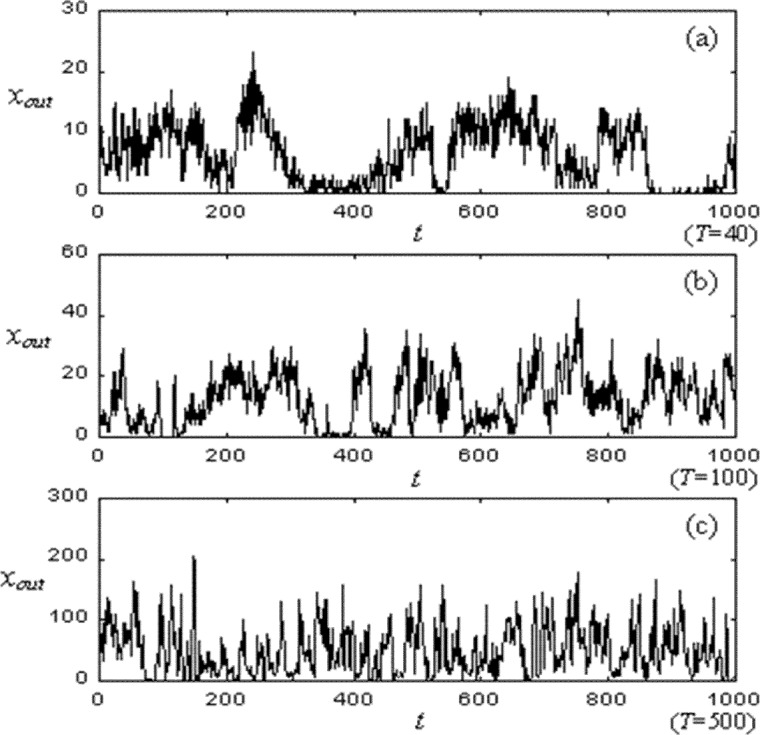
Time series of *χ*_out_ at three time granularities *T* = 40, 100, and 500.

Let *χ*_out_ denote the number of data packets received by a node, during a time interval *T*, where the node is the data packet’s destination. Measures of *χ*_out_ appear to reflect more the influence of spatial-temporal information than is the case for measures of queue size, *Nr*. Figure 4, which shows a time series of *χ*_out_ for one node at three time granularities *T* = 40, 100, and 500, supports this observation. Here, while autocorrelation decreases as *T* increases, the rate of decrease appears more gradual than is the case with queue sizes (Fig. 3). In our view, *χ*_out_ reflects more information about its time interval and about nearby space because it is an aggregate value, accumulated over the sample interval, rather than a snapshot of system state at one time instant. To gain more insight into our observations, we compute the power spectrum *S*_out_(*f*) of *χ*_out_.

**Fig. 5:**
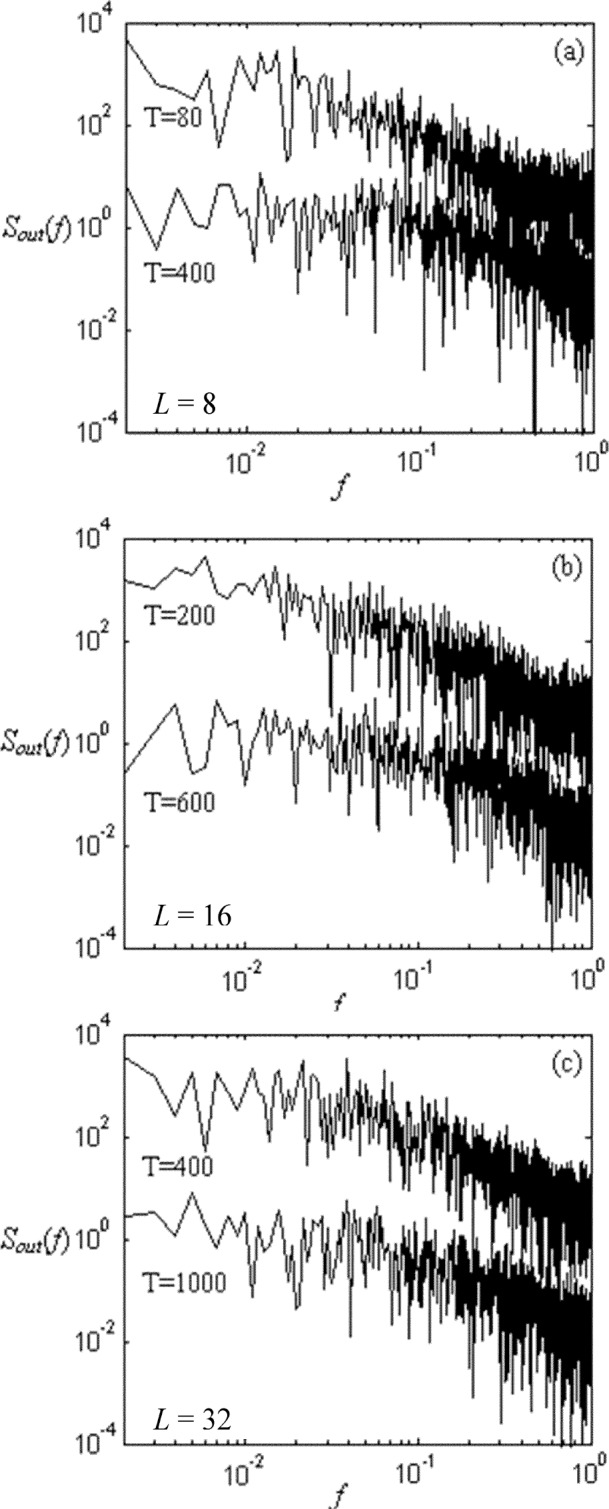
Power spectrum *S*_out_ of *χ*_out_ with (a) *L* = 8 for *T* = 80 and 400, (b) *L* = 16 for *T* = 200 and 600, and (c) *L* = 32 for *T* = 400 and 1000.

Figure 5 shows the power spectra for selected combinations of time granularity and system size. In general, each curve shows a flat line at lower frequencies, followed by a negatively sloped line commencing at higher frequencies. The negatively sloped portion of each curve exhibits the appearance of 1/*f* noise. In our interpretation of these curves, the larger the negatively sloped region of a curve (or the shorter the flat region), the more 1/*f*-like the curve. Further, the more a curve appears 1/*f*-like, the greater the long-range dependence in the signal. For example, comparing the two curves in Fig. 5(a), we find that long-range dependence decays as the time granularity increases from *T* = 80 to *T* = 400. We also find that for the same time granularity (*T* = 400), a larger network size shows a greater long-range dependence (compare Fig. 5(a) *T* = 400 against Fig. 5(c) *T* = 400). The presence of 1/*f* noise, which is characterized by correlations extending over a wide range of time scales (long-range dependence), provides a clear indication of some sort of collective effect. Moreover, using our interpretation, the graphs in Fig. 5 show that long-range dependence decays as *T* increases for the same system size, *L*, and that long-range dependence holds for the same time granularity as system size increases. This suggests that congestion dissipates more slowly as network size increases, that is, a larger network seems to have a more pronounced correlative structure. We believe such behavior occurs because network traffic experienced at a node consists of transient packet flows transiting across a mixture of short and long distances, and modulated by adaptive congestion-control algorithms.

**Fig. 6:**
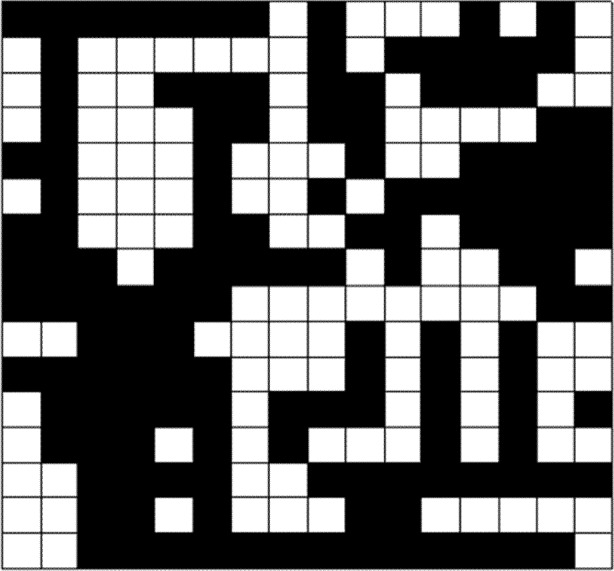
2-D map of congested network nodes.

**Fig. 7:**
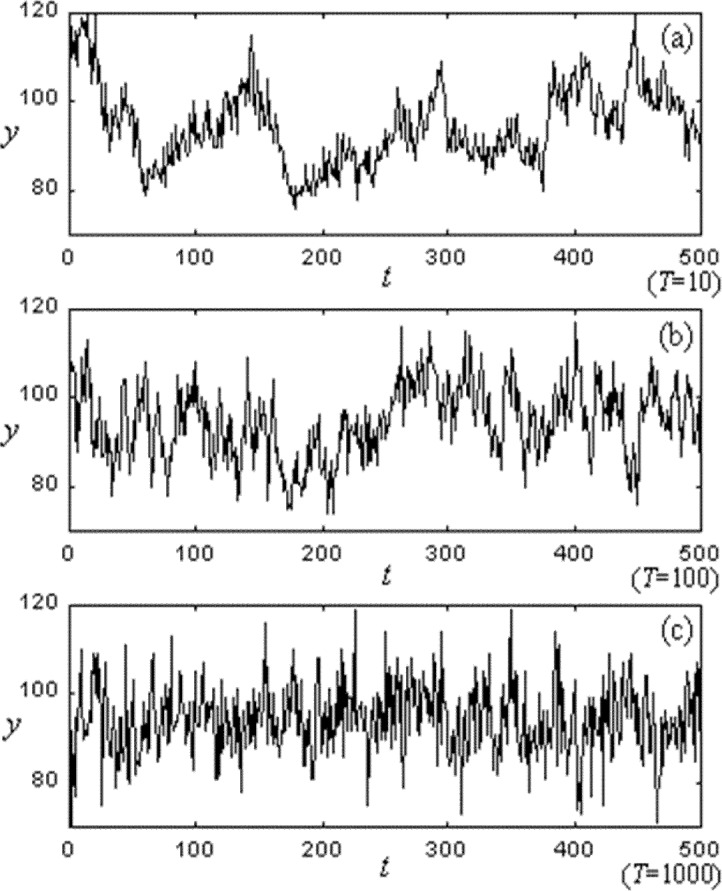
Time series of the number of congested nodes, *y*, with congestion threshold, *Y* = 5, at three different time scales: *T* = 10, 100, and 1000.

To investigate this behavior more directly, we developed a technique to monitor the congestion present in aggregate among all nodes in our network model. Our technique maps the three-dimensional (3-D) structure (*L*×*L*×*Nr*) of the network state onto a two-dimensional (2-D) binary pattern. To achieve such a mapping, we set a threshold parameter *Y* against which to compare the state (i.e., the queue length) of every node. If the queue length of the node *r* is less than or equal to *Y*, then the state value *br* of the binary network is set equal to zero, otherwise one. In this way, the network state can be mapped from three dimensions onto a 2-D grid, as shown for example in Fig. 6 where black blocks represent congested nodes (for *Y* = 5), and white blocks depict congestion-free nodes. Using such a 2-D map proves more convenient then a 3-D map when visualizing the evolution of network state. Such maps provide a more readily comprehensible view of the spatial correlation of network congestion. As time progresses, the congestion state of a node in a large network depends more on the congestion state of its neighboring area. This idea was first proposed by researchers who modeled the propagation of congestion between neighboring routers based on contact processes with a Cayley tree [10]. Using the 2-D grid, we can determine the number of congested nodes (*y*) in our model at any time granularity *T*, and then record *y* as a time-series representing the system state for any number of sample intervals *t*. In Fig. 7 we show a time series of congested nodes for a system size *L* = 16 at three time granularities: *T* = 10, 100, and 1000. As the figure shows, we find generally that as the time granularity, *T*, increases, the number of congested nodes, *y*, changes less smoothly. This suggests that congested nodes exhibit stronger interdependence at smaller time scales.

**Fig. 8:**
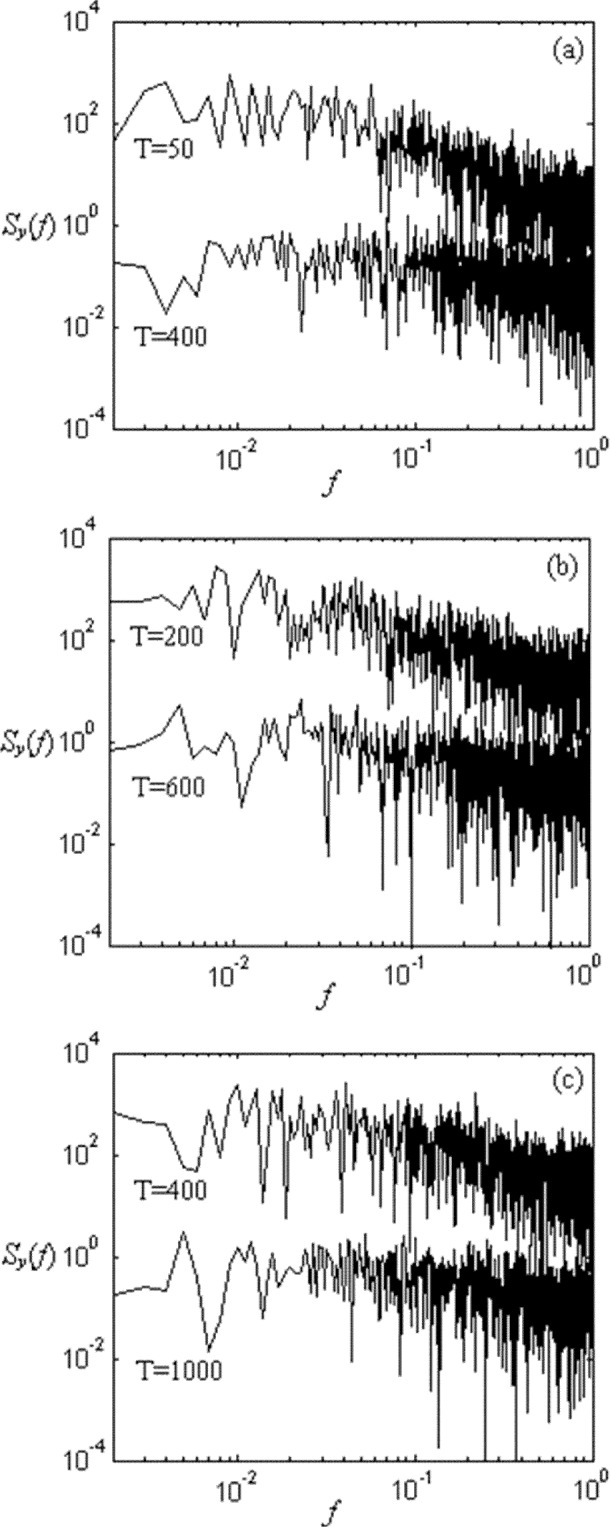
Power spectrum of *S_y_* (*f*) of *y* with (a) *L* = 8 for *T* = 50 and 400; (b) *L*= 16 for *T* = 200 and 600; and (c) *L* =32 for *T* = 400 and 1000.

To explore the relationship among time scale, network size, and congestion, we plot several power spectra in Fig. 8 at various network sizes and time granularities. Figures 8(a)–(c) illustrate that increasing the time granularity for the same network size leads to a reduced appearance of 1/*f*-like noise. For example, comparing the two curves in Fig. 8(a) (network size *L* = 8), we find some autocorrelation in the curve *T* = 50 but the curve for *T* = 400 appears almost flat, suggesting little autocorrelation in the signal. Comparing this curve for *T* = 400 against the curve for the same time granularity but at network size *L* = 32, shown in Fig. 8(c), we find that as the network size increases from *L* = 8 to *L* = 32 the long-range dependence increases for a given time granularity. To us, these results suggest that collective behavior in a larger network causes more profound influence on network congestion and on predictability. If true, these factors might prove meaningful for the design of congestion-control and traffic-engineering mechanisms in networks.

We suspect that 1/*f* noise arises from the collective effect of many interacting network flows, independent of specific details associated with network protocols. Furthermore, as indicated by Fig. 8, the collective effect appears to strengthen as system size increases, but to diminish as time granularity increases. At very large time scales, the evidence of collective effect does not appear; instead, the power spectra tend toward whiteness as the sample interval (*T*) exceeds some size. These observations suggest that some time-granularity threshold might exist within which the network can be viewed as a coherent whole (that is, a time granularity where the network congestion signal exhibits the most pronounced 1/*f*-like appearance). We can refer to this granularity as the *coherent time scale*. As shown by Fig. 8, the larger the network, the greater the coherent time scale, because 1/*f*-like noise appears at larger sample intervals. On the other hand, our results also suggest some difference in the evolution of congestion between large and small networks. In our spectral analyses, a larger network, such as *L* = 32 in Fig. 8(c), retains a more 1/*f*-like appearance at larger time scales than does a smaller network. On the contrary, as plotted in Fig. 8(a), the 1/*f*-like appearance in the congestion signal appears to decay faster as *T* increases in the smaller network (*L* = 8). To us, these results confirm that a congestion-control system or a traffic-management regime has more time available (a larger coherent time scale) to respond to congestion in a larger network, because the congestion diminishes rather more slowly than in smaller networks. Of course, the management of a large network also requires more time. The effectiveness of control methods may fall off gradually once their reaction time exceeds a certain threshold. Discussion regarding the influence of these findings on concrete network management techniques is beyond the content of this paper.

Considering the collective dynamics of a network and the relationship between network size and time granularity, we find that for a given network size one must expect more volatility in congestion as the sample interval increases. We have compared our TCP and CAC congestion-control algorithms against cases where the network does not control congestion. These latter experiments, not reported in this paper, modulate congestion by varying the intensity of source traffic. Across all of our experiments, we find that long-range dependence emerges at different time scales, seeming to depend not only on network size but also on traffic intensity and congestion-control algorithm.

**Fig. 9:**
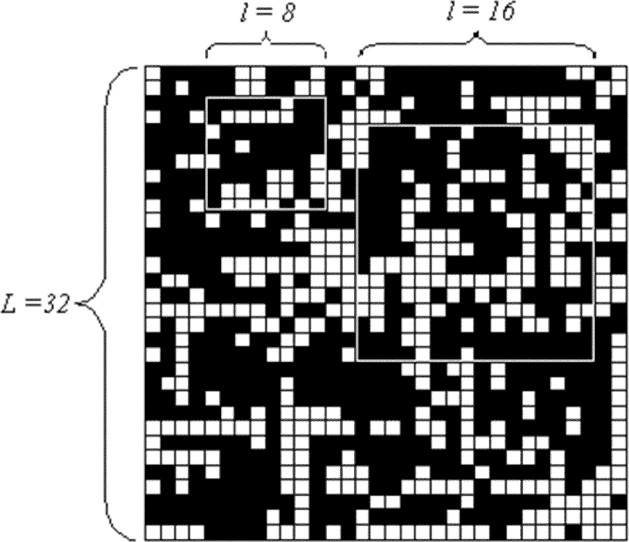
A binary pattern of network congestion in a network of size, L = 32, and identifying two sub-areas with sizes *l* = 8 and *l* = 16.

**Fig. 10:**
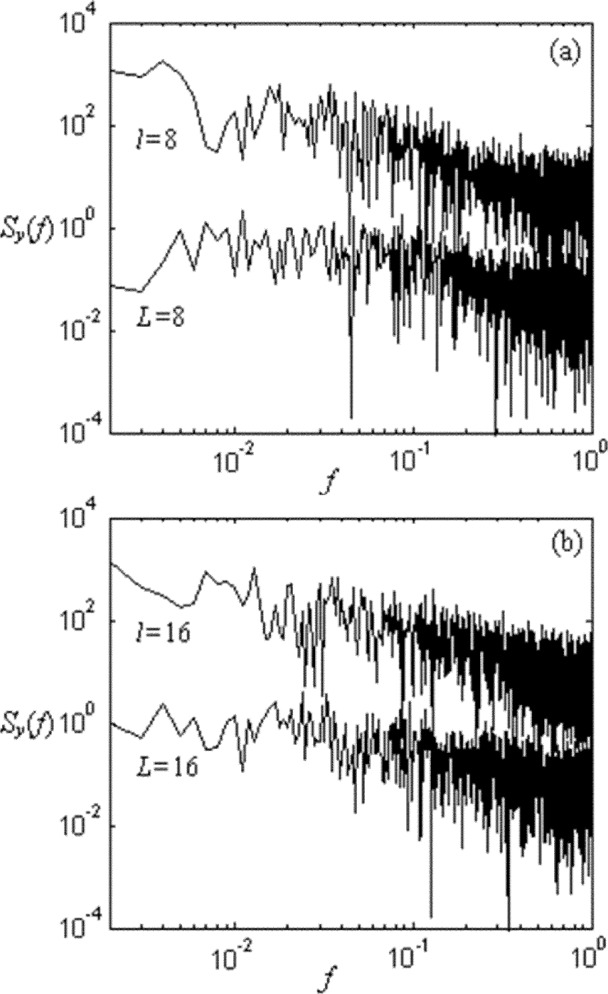
Power spectra *S_y_* (*f*) of number of congested nodes, *y*, with (a) *L* = 8 compared against sub-area, size *l* = 8, in a network of size *L* = 32 for *T* = 200 and (b) *L* = 16 compared against sub-area, size *l* = 16, in a network of size *L* = 32 for *T* = 400.

One particular phenomenon observed in our current experiments might provide some useful insight for researchers attempting to explore behavior in communication networks. Our results suggest that the congestion response of a sub-area in a larger network may have different features compared with a smaller network with the same size as the sub-area. For example, Fig. 9 illustrates a binary network pattern with *L* = 32 and indicates two sub-areas with sizes *l* = 8 and 16. Each sub-area appears as an interconnected or interwoven part of the larger network; thus, these sub-areas play an indispensable role in global emergence, where collective behavior pertains to the system as a whole. Extracting such sub-areas, by isolating them from the original network into a smaller network with system size *L* = *l*, ignores various relationships or interdependencies, and may lead to inaccurate analysis regarding some aspects of dynamic behavior in a large network. We demonstrate this effect in Fig. 10.

Figure 10(a) compares the power spectrum of the number of congested nodes, *y*, at a comparable time granularity, *T* = 200, for a network of size *L* = 8 against a sub-area of comparable size, *l* = 8, from within a larger network with size *L* = 32. Figure 10(b) provides a similar comparison, except that the time granularity is increased to *T* = 400, and the network size and sub-area size are increased to *L* = 16 and *l* = 16, respectively. These figures suggest that sub-areas exhibit stronger dependence with respect to congestion than networks of the same size and time scale. For this reason, network researchers interested in the behavior of networks of a certain size *L* would be well advised to investigate such networks as sub-areas of size *l* = *L* within a larger network. Further, these results also suggest that network researchers should strive to investigate the effects of network congestion and cross traffic within topologies exhibiting sufficiently large scale. The global congestion behavior of a network can look quite different depending upon whether a set of identical nodes is arranged in a small or large network. This might represent a caution for researchers who rely on detailed, discrete-event simulations, because simulation models can take substantial computing resources and memory as the size of the topology increases. Yet, a sizeable topology appears necessary in order to approximate the effects of congestion within even a restricted sub-area.

## 4. Discussion

We aim to understand collective dynamics in large networks, where cause-effect relationships might not be inferred readily from the behavior of individual nodes. Our current results suggest how such collective dynamics might arise and evolve. We start with a disordered network, where the nodes act in a random way (based on a memoryless input process), and where any propagating influence will be dispersed and dissipated quickly. Initially, we saw that nodes were only interacting locally. Such locality of interaction follows from the basic continuity of physical processes: for any influence to pass from one region to another it must first pass through all intermediate regions. During the time that the process propagates through the intervening regions, it will be disturbed by all the fluctuations taking place within those regions. As a result, in the network’s original disordered state, distant parts of the network do not influence each other: they appear independent. Over time, as feedback control mechanisms adapt to changes in network congestion in all directions, a discernable structure increases gradually and then continues to evolve. Eventually, the discernable structure expands into a global order. These effects depend upon the size of the network, appearing more strongly over greater time scales as network size increases.

No approach to simulation can describe the behaviors of the real Internet completely. Given the state-of-the-art, it is currently within reach to develop good temporal models to study performance at a single node or in a small network (i.e., to represent a smaller part of a larger network). We believe we have shown that it would be desirable, and perhaps feasible, to devise models to study the spatial-temporal performance of a large network as a whole. To achieve such an outcome requires that simplifications be made. While our modeling method seems an extreme simplification, especially in terms of the regular topology, the homogeneous links and nodes, and the routing of traffic, we do maintain the individual identity of packets and we believe that our model captures important details, such as feedback congestion control, that might be missed in other network simulations. Our model enables us to explore collective dynamics in reasonably large networks, up to 1024 nodes so far. Our model also drives 2-D and 3-D animations (not described in this paper) that give an intuitive view of network behavior over time and against key parameters.

## 5. Future Work

The results reported in this paper, as well as elsewhere [8, 9, 14–16], encourage us to continue our investigations into the collective dynamics of large networks. We foresee three directions in our immediate future plans. First, we need to incorporate into our network model additional traffic sources and control mechanisms related to providing quality of service. As more and more high-speed access links are added to the Internet, the nature of traffic can be expected to change, perhaps leading to an increasing quantity of streaming multimedia traffic and large file transfers, as well as increases in traffic from various interactive group games and from instant messaging applications. In addition, the pattern of source-destination pairs may evolve as users begin to move toward increased peer-to-peer communications. In addition to changing traffic patterns among network nodes, evolving Internet use might alter the balance between TCP and non-TCP traffic. Such changes will increase the criticality of deploying mechanisms to provide quality of service. While researchers have provided an understanding of the properties of various quality-of-service mechanisms on a local scale, the effects of such mechanisms have not been studied on a global scale in large networks.

Second, we must increase the size of our model in order to improve our ability to identify and understand emergent phenomena, and especially congestion dynamics, at Internet scale. The current work reports a maximum network of 1024 nodes (*L*×*L* = 32×32= 1024) and 4096 links (four links per node). Using MATLAB, we were able to model a network of this size; however, to execute the behavior of the network for two million time steps requires 10 days of CPU time on a 750 MHz Pentium III with 128 MB of memory. We intend to recode our model using C or C++ in an effort to achieve a network size on the order of 16 000 nodes and 64 000 links. Such an increase in size should enable us to enhance our ability to identify and understand emergent phenomena. To reach our ultimate intended network size, on the order of 10^5^ to 10^6^ nodes and links, we might require a parallel processing system. Such systems appear well suited to cellular automata [23].

With a network model of sufficient size, we can undertake a systematic search to identify, understand, and perhaps explain, significant emergent phenomena in large networks. This represents our third avenue for future work. In this case, we must develop theories regarding the most likely emergent phenomena, and then test those theories with a systematic set of experiments. From this work, we might well provide guidance to researchers seeking to measure Internet behavior on a global scale. In particular, we might identify specific phenomena and related measurement data that could be collected by experimenters in order to confirm or refute the presence of emergent phenomena within the Internet.

## 6. Conclusions

When attempting to analyze network behavior by examining the behavior of constituent components, researchers can develop the misconception that uncertainty regarding network congestion and resource consumption increases with network size. We argue that the opposite holds. We find that correlation in congestion among network nodes can be expected to persist at larger time granularities as system size increases, and we also find network size and time granularity to be two closely related aspects of the spatial-temporal dynamics of a network. This suggests that as network size increases, the collective behavior of a network might well become more predictable. Such predictability arises from global emergence, where chain reactions move through the whole system. As the system becomes larger, the correlated interactions persist. As we show, such collective behavior cannot be realized from the analysis of individual network components.

Although in this paper we do consider two simplified congestion-control algorithms, we must still evaluate the collective dynamics of large networks that include additional mechanisms, such as differentiated services and routing updates. However, more important challenges revolve around issues of scale. We need to scale the network model up to a million nodes, so that we can investigate multi-scale, spatial-temporal dynamics. This suggests a need to employ a parallel programming environment to study multiple-timescale traffic patterns and network performance, and to identify and understand even more interesting phenomena arising only in sufficiently large networks. We believe that simulating networks at an appropriately large size is key for researchers to gain insight regarding behaviors that might emerge within the Internet.
